# Prevalence and determinants of poor glycaemic control in individuals aged between 18-60 years, at a regional hospital in KwaZulu-Natal Province, South Africa- a cross sectional study

**DOI:** 10.4314/ahs.v23i4.36

**Published:** 2023-12

**Authors:** Lindelwa Zimu, Ozayr Mahomed

**Affiliations:** University of KwaZulu-Natal College of Health Sciences, Public Health Medicine

**Keywords:** Diabetes mellitus, poor glycaemic control, regional hospital, optimising treatment, prevalence, determinants

## Abstract

**Background:**

Achieving optimal blood glucose control is imperative for preventing diabetes related complications and negative socio-economic consequences associated with them.

**Objectives:**

The aim of the study was to determine the prevalence and determinants of poor glycaemic control amongst type II diabetic outpatients presenting at a regional semi-rural hospital in eThekwini district, Kwa-Zulu Natal.

**Methods:**

An observational, analytic cross-sectional study was conducted amongst 384 systematically sampled type 2 diabetes patients. Data were collected by an interviewer administered questionnaire, clinical record review and anthropometric measurements. Bivariate and multivariate analyses were performed

**Results:**

Ninety one percent of the study population (349/384) had poorly controlled diabetes. Amongst uncontrolled diabetics, 80% (n=281) were older than 35 years' age group; 58% (n= 203) were male; 85% (n=295) completed primary school education and 93% (n=324) were overweight. Patients that were 35 years and older, female, employed, had a high body mass index, were on oral hypoglycaemic and/or insulin in combination, and receiving treatment longer than 3 years, had an increased odd of uncontrolled diabetes. Being female and receiving oral hypoglycaemic and/or insulin were significantly associated with poor blood glucose control.

**Conclusion:**

Patient that were female overweight, having a lower level of education, and greater than three-year duration of medication and on oral hypoglycaemic agent and/or insulin were more likely to have poor blood glucose control. These factors should serve as early identifiers of potential poor control and an alert clinician to adopt a more active approach to optimize treatment.

## Introduction

Diabetes is a rapidly growing global public health challenge of the 21st century associated with rapid urbanisation, globalisation, unhealthy diets, increasing sedentary lifestyle as well as an ageing population.^1^,^2^ The International Diabetes Federation in 2017 estimated that the diabetic population in Africa will increase from 16 million to 45 million by the year 2045, 2.56 times increase. An estimated 1.83 million people (5.4%) were living with diabetes in 2017. 3 Mortality due to diabetes has been steadily increasing in terms of overall rank in cause of mortality. Diabetes was ranked as the 5th leading causes of death in South Africa during 2011 to 2013, third in 2015 and the second most common natural cause of death in 2016, being responsible for 5.5 percent of deaths.^4^

Poorly controlled diabetes does not only result in death or a diminished quality of life however it may cause diabetic complications and socio-economic consequences that not only affect the individual but the community and health system at large.^5^ Sub-optimal diabetes control can result in short and long term, micro and macro-vascular complications such as, nephropathy, cardiovascular disease, neuropathy and eye damage (diabetic retinopathy and cataracts).^6^ Diabetic complications cause financial losses to the patient and the patient's family through loss of employment^7^, increased cost for transport as well as the cost of insulin and other medications.^8^

Uncontrolled blood glucose also causes losses to the health system and national economy due to healthcare expenditure.^7^ Diabetes impacts the economy due to increased use of health services and loss of productivity as a result of diabetes related complications.^8^ At a health system level, the global health expenditure for treating diabetes and preventing complications has been estimated to range between 673 million to 1.197 billion US dollars in 2015 and projected to increase to more than 802 million to 1.452 billion US dollars.^7^ Estimates from the International Diabetes Federation 2019 indicate that South Africa spent approximately $5.707 million dollars for diabetes related care in 2018 or an estimated mean expenditure of $1245 per diabetes patient. 9

A study to assess the determinants of glycaemic control among patients with Type 2 diabetes mellitus (T2DM) presenting at the Greater Accra Regional Hospital, Ghana in 2017 indicated that high body mass index (BMI) and use of insulin therapy were associated with poor glycaemic control (high HbA1c) whereas low levels of coffee intake were positively associated with glycaemic control. Self-monitoring blood glucose and diet therapy did not appear to influence glycaemic control. When age and gender were controlled, high physical activity was positively associated with blood glucose control^10^. A Mixed-method systematic review of the literature published between 2006-2017 was conducted using seven electronic databases (CINAHL, MEDLINE, Scopus, EMBASE, PsycINFO, PubMed and ScienceDirect) suggested that higher socio-economic status, greater dietary knowledge, and higher self-efficacy and empowerment improve glycaemic control among patients with diabetes mellitus^11^.

The global report on diabetes 2016 by the World Health Organisation 12 identified the following as factors from a health system perspective that are associated with uncontrolled blood glucose: delayed diagnosis of type II diabetes, difficulty in accessing treatment and basic diagnostic materials, poor referral and back referral systems and a lack of medications. In addition, blood glucose control can also be hindered by: a lack of knowledge of healthcare workers on how to define good blood glucose control, insufficient monitoring of blood glucose control, difficulty in managing elevated blood glucose, the need for intensive treatment and insufficient specialist diabetes care units^13^.

The recommended glycated haemoglobin (HbA1c) target to prevent microvascular complications and macro-vascular complications when intensive treatment is instituted in the course of the disease is <7%. For newly diagnosed patients in good general health and reasonable life expectancy an HbA1c target of <6.5% is reasonable to aim for to further reduce microvascular risk, provided it can be achieved safely.^14^

Controlling non-communicable diseases (NCDs) is a key priority area for the South African government and one of the targets of the Department of Health is to increase the percentage of people with controlled hypertension, diabetes and asthma by 30% by 2020 in sentinel sites. 15 There are a paucity of studies on the prevalence and determinants of poor blood glucose control in our local context.

The aim of this study was to assess the prevalence and determinants of blood glucose control among type 2 diabetes outpatients aged between 18-60 years, receiving treatment at a regional semi-rural hospital.

## Research methods and design

### Study design

The overall study was a cross sectional study with data collected via a retrospective chart review and patient survey.

### Study setting

Prince Mshiyeni Memorial Hospital (PMMH) is a regional hospital situated in the 2nd most densely populated township in South Africa known as Umlazi. The hospital is a 1200 beds hospital facility and receives patients on a referral basis from surrounding primary health clinics (PHC) for specialized management of diabetes and other illnesses.

### Study population

The inclusion criteria were confirmed cases of type 2 diabetes (T2D) aged 18 to 60 years, ambulatory patients who have been receiving diabetic medication for a minimum of 1 year. Both controlled ((HBA1c <7%). and uncontrolled persons living with diabetes (PLW) (HBA1c >7%). were included in the study.

### Sample Size and Sampling strategy

We calculated the minimum sample size of participants using a single population formula, by taking 50% of population, 80% power and 95% confidence interval and determined the required sample to be 384. To identify participants a systematic random sampling method was used. There is no register of all the patients visiting the hospital on any particular day. The number of patients that present at the diabetic clinic at PMMH differs each day. A random starting point was selected after which the fifth person in the queue waiting to be seen by the doctor was the starting point and thereafter every fifth patient was selected for the study.

### Data Collection

Data were collected from February 2018 to September 2018, between 07:00 and 11:00 on weekdays. The patients HbA1c level and presence of co-morbidities were extracted from clinical records. The Anthropometric measurements (weight and height) for each patient were taken using a stadiometer and a calibrated scale prior to completion of the questionnaire. A questionnaire was then administered by the researcher, in the language that the patient felt most comfortable with, which was either English or Zulu. The questionnaire was designed to collect the following information: sociodemographic details, (includes as gender, age, marital status, level of education and employment status), lifestyle factors: adherence to treatment, support structure, knowledge regarding uncontrolled diabetes and its management as well as health care delivery factors.

### Data processing and analysis

Double entry of data was performed using the Epi Info software tool by a data capture to 1041 ensure and exported to Statcorp Software for Statistics and Data Science (STATA) version 13 for analysis. Descriptive variables were summarized using frequency distributions and a pie chart was used to present the prevalence of uncontrolled diabetes Bivariate and multi-variate logistic regression were used to assess the influence of various variables on blood glucose control and presented using Odds Ratios (ORs) with its 95% confidence intervals (CIs). A p-value <0.05 was used for statistical significance.

### Ethical consideration

The study was approved by the Biomedical Research and Ethics committee (BREC) of University of KwaZulu Natal (BE 011/17). Permission was also obtained from the Kwa-Zulu Natal Department of Health and from the medical manager and management at Prince Mshiyeni memorial hospital. The methods were carried out in accordance with the relevant guidelines and regulations. Written informed consent was obtained from all participants. Participants were informed about their rights to refuse or withdraw, and confidentiality of the individual information obtained.

## Results

### Sociodemographic and clinical characteristics

Three hundred and eighty-four (type 2 diabetes patients participated in the study. Eighty percent (309) were above the age of thirty-five. More than half, 229 (59%) of the participants were females. Sixty-two percent (n=239) participants had some form of employment (self-employment, temporary or permanent employment) whilst were 37% (144) were either unemployed or receiving a pension. The mean body mass index (BMI) in the study population was 31.1kg/m^2^, 92% (n=354) had a BMI greater than or equal to 30kg/m2. Approximately 59 %(n=229) of the participants have been living with diabetes for 3 years or more. Seventy-three percent (n=281) patients were on oral medication. Ninety one percent (352) of the study participants had two or more co-morbidities (Table 1).

**Table 1:** Sociodemographic and clinical characteristics of type 2 diabetes outpatients at a regional hospital in KwaZulu Natal-2018

Category	Subcategory	Glycaemic control				Study Population		p-value
		Good (n)	Good (%)	Poor (n)	Poor (%)	Total (n)	Total (%)	
Sex	Female	27	75	203	58	230	59	0.05*
	Male	9	25	146	42	155	41	
Age group	18- 35 years	8	22	68	19	76	20	0.69
	35 years and above	28	77	281	81	309	80	
Level of Education	Low Level	27	75	295	85	322	84	0.14
	High level	9	25	54	15	63	16	
Support at home	Yes	3	9	52	15	329	85	0.28
	No	33	91	297	85	56	15	
Employment	Employment (self, temp, permanent)	11	31	135	39	239	63	0.34
	Pensioner/no employment	25	69	214	61	144	37	
Duration of diabetes	< 3 years	16	44	140	40	156	41	0.61
	> 3 years	20	56	209	60	229	59	
Type of medication	Oral	31	86	250	72	281	73	0.06
	Insulin	5	14	99	28	104	27	
Presence of two or more comorbidities	Yes	31	14	321	92	352	91	0.231
	No	5	86	28	8	33	9	
BMI (kg/m2)	Mean (SD)	30.19 (0.96)		31.25 (0.32)		31.15 (0.31)		0.84

Amongst uncontrolled diabetes patients, 80% (n=281) were above 35 years' age group; 58% (n= 203) were female; 85% (n=295), had a lower level of education. 93% (n=324) were classified as obese, 60 %(n=209) had been diagnosed with diabetes for a period of 3 years or more; 72 %(n=256) were on oral medication and 92% of the participants had two or more co-morbidities (Table 1).

### Predictors of poor glycaemic control

Ninety-one percent (n=349) of the study population had uncontrolled blood glucose levels (HBA1c >7%). Bivariate analysis showed that the age group (35-60 years and above) (OR: 1.18 CI: 0.44-2.80), being female (OR: 2.15 CI: 0.94-5.36); employment (OR: 1.35 CI: 0.65-3.33) and a BMI >25 kg/m2 were associated with an increased odd of having poorly controlled blood glucose. In addition, patients on oral medication plus insulin (OR: 2.45, CI: 0.90-8.30), having diabetes for longer than three years (OR: 1.19 CI: 0.55-2.51) and having two or more co-morbidities 1.84(CI: 0.51-5.33) associated with an increased odd of having poorly controlled blood glucose. After multivariate analysis, female gender; (OR: 2.55 CI: 1.11-5.84), and patients on oral medication and/or insulin versus patient receiving oral medication (OR: 3.69, CI: 1.19-11.36) was significantly associated with poorly controlled type 2 diabetes (Table 2).

**Table 2:** Sociodemographic and clinical Predictors for poor glycaemic control in patients with type 2 diabetes, at a regional hospital in KwaZulu Natal-2018

Variable	Bivariate analysis			Multivariate analysis		
	Unadjusted Or	P-Value	95% Ci	Adjusted Or	P-Value	95% Ci
Age (<35 years vs >35 years)	1.18	0.69	0.44-2.80	0.83	0.73	0.30 -2.32
Gender Female vs male	2.15	0.04*	0.94-5.36	2.55	0.02*	1.11 -5.84
Employment (Employed vs Unemployed)	1.43	0.33	0.65 - 3.33	1.75	0.17	0.78 – 3.95
Education (High school versus primary school)	0.54	0.14	0.23 -1.40	0.34	0.03*	0.132 -0.90
BMI (≥ 24.9kg/m^2^ versus <24.9kg/m2 vs	2.16	0.13	0.60 – 6.32	3.25	0.21	0.51 – 20.6
Need for support (Yes, vs No)	1.92	0.28	0.57 – 10.15	1.99	0.36	0.44 – 8.89
Duration with Disease > 3 years vs < 3 years	1.19	0.61	0.55-2.51	0.16	0.17	0.12-2.26
Duration on Medication > 3 years' vs < 3 years	1.33	0.40	0.62-2.81	7.59	0.12	0.57-99.9
2+co-morbidity versus no co-morbidities	1.84	0.51	0.51-5.33	0.92	0.93	0.14-5.70
Oral medication plus insulin versus oral medication	2.45	0.06	0.90-8.30	3.69	0.02*	1.19-11.36
Need for Support (Yes vs No)	1.92	0.28	0.57-10.15	1.99	0.36	0.44-8.89

## Discussion

The two main objectives of this study were to assess the prevalence of uncontrolled blood glucose levels and the predictors of blood glucose control among type 2 diabetes outpatients aged between 18-60 years, receiving treatment at a regional semi-rural hospital. This study shows that the overall prevalence of uncontrolled diabetes (91 %) based on an HbA1c cut-off of >7%. 16 Females, overweight patients (BMI ≥24.9kg/m^2^), and patients with diabetes > 3 years duration and being on oral hypoglycaemic and/or insulin were at an increased odds of poor diabetes control. However, being female and patients on oral hypoglycaemic and/or insulin were statistically significant.

Poor glycaemic control was defined as glycated haemoglobin (HbA1c) >7% in accordance with the guidelines of the Society for Endocrinology, Metabolism and Diabetes of South Africa and this is a higher cut off point for the HbA1c target as HbA1c target is <7 % to prevent microvascular complications, and macrovascular complications 16 and our findings are therefore very concerning. The overall prevalence of uncontrolled blood glucose level of 91% exceeds the findings from a previous studies in South Africa - OR Tambo district, in the Eastern Cape of South Africa (83.8%) 17, a regional hospital in KwaZulu Natal (83%) 18 and a provincial hospital in the North West Province of South Africa (69.3%).^19^ In addition, the prevalence of poor diabetes control in the current study is higher than the results of a cross-sectional study, in the rural areas of the Eastern Cape that showed the overall prevalence of poor glycaemic control was 77.71% (n = 122), while very poor glycaemic control occurred in 50.6% (n = 80) of the study cohort.^20^ Studies conducted in Ethiopia^21^, Saudi Arabia^22^, Eastern Sudan^23^, Korea^24^ all reported a prevalence of uncontrolled type 2 diabetes in the range of 70.9% - 74%. The lowest level of uncontrolled type 2 diabetes that was identified was from a study conducted in Malaysia^25^, Niger Delta^26^ and Turkey^27^ where the prevalence of uncontrolled type 2 diabetes ranged from 38.4% up to 67%. The higher-than-expected level of uncontrolled diabetes in our study is influenced by the study sample as the patients attending the hospital are patients that have poor glucose control and are referred for blood glucose control to the hospital.

In our study we extracted HbA1c levels for 2018 year, as most patients were on treatment for greater than one year. HbA1c levels at diagnosis will have skewed the prevalence towards a greater proportion of patients being uncontrolled.

Sixty five percent (250) of the patients with poorly controlled diabetes were on oral hypoglycaemic agents. Oral hypoglycaemic agents and/or insulin were significantly associated with poor glucose control versus oral hypoglycaemic combination. These findings are similar to results from his cross-sectional study that drew participants from over 15 community health centres (primary care centres) in OR Tambo district, South Africa, to Mthatha General Hospital between June and November 2013 that showed the risk of poor glycaemic control was higher among those on a combination therapy of insulin and metformin and a higher risk of poor glycaemic control was found among patients on insulin) in comparison with those without insulin^17^.

Although, not to the same magnitude systematic review of the Gulf Cooperation Countries (GCC) reported that odds of poor control was 2.64 times higher for patients on insulin compared to participants on diet regimen only.^28^ A number of other studies showed poor glycaemic control to be significantly higher amongst patients that were on insulin therapy or insulin and oral anti-diabetics compared to those on oral medication only.^27^ This is further corroborated by a cross-sectional study conducted in Ethiopia that showed patients that used combined insulin and oral medication were approximately five times (OR= 4.59, p-value0.04) more likely to have poor glycaemic control.^21^

Initial management for diabetes is through education on an appropriate diet and encouraging exercise, thereafter followed by the addition of an oral hypoglycaemic medication, then an increase in dosage and the addition of combination therapy as a final resort. The findings in our study may be attributed to a lag in consultation between referral and consulting a doctor as well as delayed initiation of combination therapy as a result of some level of clinical inertia, where physicians might be slow in responding to the clinical parameters. 29

Females were at an increased the odds of poor blood glucose control (OR: 2.15 p-value <0.05). Similar findings were shown in the Eastern Cape study.^17^ Furthermore our findings are consistent with a similar study cross-sectional study in Shannan Gibe, South West Ethiopia in 2013 that reported the odds of poor glycaemic control were 1.58 times higher for females compared to males, although not statistically significant.^30^ The association between gender and blood glucose control remains unclear due to conflicting results found in other studies. Only one reported of the ten studies from a systematic review conducted in the Gulf Cooperation Council countries (GCC) reported poor glycaemic control was associated with females and poor glycaemic control. 28 In addition, a study conducted in Jizan City, Saudi Arabia did not find any association between gender and HbA1c level^22^, whilst a cross-sectional study conducted in June 2014 amongst 292 type 2 diabetics in Ethiopia showed that males had an increased odds of poor glycaemic control (COR=2.34, AOR=3.47). 31

A number of potential factors contribute to the increased odds of females having poor blood glucose control. Females are responsible for increased workload that may hinder their compliance to medication. Furthermore, the high unemployment rate may hinder access to health facilities. Compounding these factors is the triple burden of inadequate physical activity, obesity and high cholesterol that increased their risk for poor control.

## Limitations of the study

This study was a cross sectional design study and therefore a cause-and-effect relationship between type two diabetes and the determinants of poor glucose control could not be established. The questionnaire was self-administered and recall bias for adherence, support, and level of education cannot be ruled out. This may have resulted in some recall bias. The study was conducted only amongst patients who were on follow up at the diabetes clinic at a hospital may have over-estimated the level of poor control.

Recent studies have questioned the use an HbA1c value alone as a therapeutic goal in the absence of information regarding the relationship between HbA1c and mean glucose in each individual.^32^ Glycated haemoglobin concentration can be affected by a variety of genetic, hematologic, and disease-related factors, and the specific effects depend on the specific haemoglobin variant or derivative and conditions influencing RBC lifespan.^33^-^35^ HbA1c values are inappropriately low if RBC lifespan is short (e.g., in haemolysis) or if RBC age is low (e.g., in acute blood loss), and are inappropriately high in iron deficiency anaemia, although this can be corrected by iron supplementation. These factors may have influenced the HbAlc levels in our study population resulting in an over-estimation of poor blood glucose control.

## Conclusions

This study shows a very high prevalence of poorly blood glucose levels. Being female and being on oral hypoglycaemic agent and/or insulin were the most significant predictors of poor diabetes control. Although, being overweight, having a lower level of education, and greater than three-year duration of medication showed increased but not significant odds for poor diabetes control, it is imperative for clinician to consider these underlying determinants in overall management of patients. There is a need for early identification of patients at risk for poor control and a more active approach by clinicians to optimize treatment to prevent long term complications.

## Data Availability

The datasets used and/or analysed during the current study are available from the corresponding author on reasonable request.

## Abbreviations

BMI: body mass index
CIs: confidence intervals
HbA1c: glycated haemoglobin
NCDs: non-communicable diseases
OR: Odds ratio
PMMH: Prince Mshiyeni Memorial Hospital
PHC: primary health clinics
STATA: Software for Statistics and Data Science
T2D: type 2 diabetes
